# Population Dynamic of the Annual Halophyte *Salicornia ramosissima* in Salt Pans: Towards a Sustainable Exploitation of Its Wild Populations

**DOI:** 10.3390/plants11131676

**Published:** 2022-06-24

**Authors:** Alejandro Polo-Ávila, María D. Infante-Izquierdo, Enrique Sánchez-Gullón, Jesús M. Castillo, Adolfo F. Muñoz-Rodríguez

**Affiliations:** 1 Departamento de Ciencias Integradas, Fuerzas Armadas Ave., Campus El Carmen, Universidad de Huelva, 21071 Huelva, Spain; alejandro.polo@hotmail.es (A.P.-Á.); mariloli.infante@gmail.com (M.D.I.-I.); adolfo.munoz@dbasp.uhu.es (A.F.M.-R.); 2 Paraje Natural Marismas del Odiel, Ctra. del Dique Juan Carlos I, Ap. 720, 21071 Huelva, Spain; enrique.sanchez.gullon@juntadeandalucia.es; 3 Departamento de Biología Vegetal y Ecología, Universidad de Sevilla, Ap. 1095, 41080 Sevilla, Spain

**Keywords:** air temperature, inland salt marshes, Odiel Marshes, plant density, saline agriculture, salinity, salt marsh

## Abstract

Halophyte species growing under stressful conditions, such as the annual species of the *Salicornia* genus, have been recognized as a source of metabolites of pharmacological and nutraceutical interest. Therefore, planning the extraction of individual plants from wild populations in a sustainable way is especially important in the case of annual species. We studied the environmental matrix and population dynamic of four *Salicornia ramosissima* populations growing at two elevations in salt pans under a Mediterranean climate. In elevated areas, *S. ramosissima* populations presented maximum plant densities of between 628–6288 plants m^−2^ that remained almost constant until fruiting. In contrast, populations in depressed zones presented five-times greater soil-seed-bank densities and maximum plant densities than populations in elevated zones. In this context, populations in depressed zones lost c. 60% of their maximum plant densities from the end of spring and through summer. In whatever way the environmental matrix seemed to control the population dynamic of *S. ramosissima* in depressed zones, the effects of a stressful environment would interact with plant densities. In this sense, we recorded the density-dependent mortality for the densest population (max. 51,558 plants m^−2^). Our results are useful for planning a sustainable harvesting of natural populations of *S. ramosissima*.

## 1. Introduction

Halophytes are salt-tolerant plants that colonize saline environments such as salt marshes where they play a key ecological role, for instance, promoting ecosystem structuring and ecological succession [1]. In addition, halophyte species growing under stressful conditions have been recognized as a source of metabolites of pharmacological and nutraceutical interest [2,3,4]. In this context, the exploitation of wild populations of halophytes offers a great opportunity as a form of saline agriculture [5], yet halophyte preservation is threatened by several anthropogenic impacts, including unsustainable exploitation of their wild populations [6]. Therefore, sustainable management plans need to be designed for the exploitation of wild populations of halophytic species in order to avoid irrational gathering.

Most halophytes are perennials, but some halophyte species are annuals [7]. Planning the extraction of individual plants from wild populations in a sustainable way is especially important in the case of annual halophyte species, since their populations are generated each year from pre-existing seed banks [8]. To our knowledge, no previous study has analyzed the population dynamic of annual halophyte species to develop sustainable management practices.

The Amaranthaceae family (formerly known as Chenopodiaceae) presents many species that are well known as plants of pharmacological and nutraceutical interest [9]. One of its most popular genera is *Salicornia* L. that includes annual succulent halophyte plants whose stems and seeds are used in fresh salads, bread or tea [10,11], and as a source of nutraceutical and pharmacological compounds [12,13,14,15]. Specifically, *Salicornia ramosissima* J. Woods colonizes European and North African salt marshes, occurring in a wide range of habitats [16], including salt pans, since their seeds are able to germinate at hypersalinity [17,18]. The seed production of *S. ramosissima* mainly depends on plant density rather than on the number of seeds produced by each individual plant and its soil seed bank, which is drastically reduced, even totally depleted, during the first year after seed dispersal [8], as has been reported for *Salicornia europaea* L. [19].

The survival of annual *Salicornia* populations has been investigated in coastal and inland salt marshes [19,20,21]. Plant density for *Salicornia* species varies greatly among populations and is regulated by a combination of density-dependent seed production and density-independent mortality [19,22,23,24]. Thus, *Salicornia* plants grow in dense populations that could reach densities greater than 100,000 m^−2^, but most studies carried out in North European marshes indicated that although density-dependent intraspecific competition can reduce the growth of *Salicornia* plants, high plant densities did not increase mortality [16]. Only [25] found density-dependent seedling mortality in populations of *S. ramosissima* growing under an *Arthrocnemum macrostachyum* (Moric.) Moris canopy in salt pans under a Mediterranean climate, where environmental conditions were less stressful than in open salt pans and where mortality was not related to plant density. In this context, soil salinity, waterlogging, the mechanical impact of tidal action, burial by sediments and predation are among the main environmental factors determining the death of *Salicornia* plants [16,20,26,27,28,29]. In the stressful environment of salt marshes, *Salicornia* populations exhibit high levels of phenotypic plasticity, genetically fixed differences in growth phenology [30] and local adaptation to their habitats [23,31,32]. Thus, individual populations of *Salicornia* may be highly sensitive to elevation variations in salt marshes, as populations from lower areas are more tolerant of prolonged submergence and waterlogging [16], while populations in upper areas are exposed to a higher risk of drought [20]. In this sense, *Salicornia europaea* have been described as less tolerant to drought than the perennial *Sarcocornia fruticosa* (L.) A.J. Scott. [33]. Although in some populations of *S. ramosissima* outbreeding by wind pollination is not completely avoided due to the existence of protandry [34], *Salicornia* species are seen as selfing species whose populations can be considered homozygous lineages with low genetic diversity [16,35,36].

Regardless of the considerable ecological and socioeconomic interest in *Salicornia* species, the vast majority of studies on their population dynamic were carried out exclusively on the North Atlantic coasts of Europe and North America despite the observed variability that exists between species, populations and habitats [16]. In the present work, we studied the environmental matrix and population dynamic of four *S. ramosissima* populations, from seedling establishment to fructification, growing at two elevations in salt pans under a Mediterranean climate. Our first hypothesis was that plant survival within a population would be density-independent and, secondly, we hypothesized that mortality would be governed by environmental factors affecting populations differently depending on their elevation. Our results are useful for planning sustainable exploitation strategies of wild populations of *Salicornia* under the stressful conditions of a Mediterranean climate, especially in the present climate-change scenario.

## 2. Results

### 2.1. Meteorological and Sedimentary Environment

**Figure 1:**
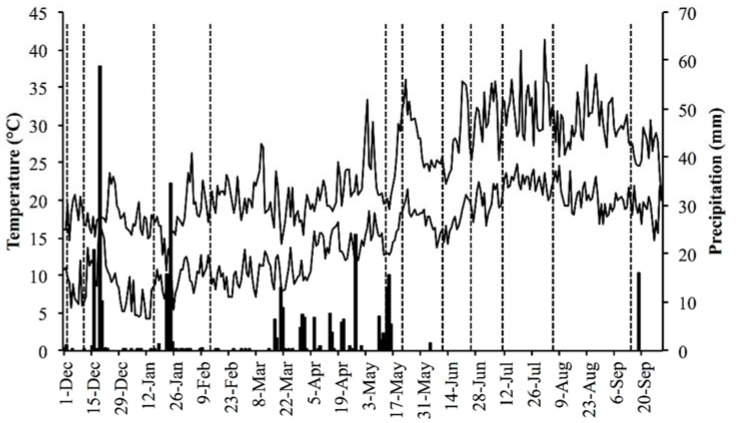
Daily maximum and minimum air temperatures (°C) (lines) and precipitation (mm) (columns) from December 2019 to September 2020 in the Odiel Marshes. Vertical dashed lines indicate sampling dates.

The mean air temperature was 19.4 °C, the mean maximum temperature was 24.1 °C and the mean minimum was 14.7 °C from December 2019 to September 2020. The rainfall was 345.0 mm during the study period (Figure 1).

**Figure 2:**
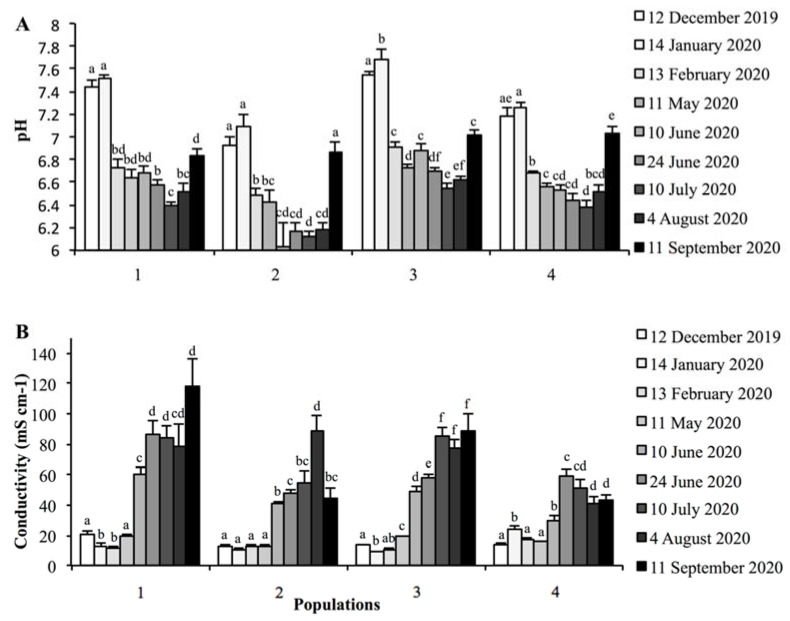
Sediment pH (**A**) and electrical conductivity (mS cm^−1^) (**B**) for four *Salicornia ramosissima* populations from December 2019 to September 2020. Different letters indicate significant differences between dates for the same population (Mann–Whitney test, *p* < 0.05). Values are mean ± SE.

The sediment pH varied between 6.5 and 7.0 in different *S. ramosissima* populations, being 3% higher in the elevated zones than in the depressed zones (Table 1 and Table 2). The sediment pH reached its maximum in January and its minimum in June–July for all populations (Figure 2A, Table 2).

The mean annual sediment EC varied between 33 ± 2 and 55 ± 5 mS cm^−1^, with no significant differences between physiographic zones (Table 1 and Table 2). The lowest values of sediment EC were recorded during winter and the highest values in summer for all populations (Figure 2B, Table 2).

### 2.2. Soil Seed Bank and Population Dynamic of Salicornia ramosissima

The seed-bank density of *S. ramosissima* varied between 7671 ± 1767 seeds m^−2^ for P2 and 124,620 ± 30,825 seeds m^−2^ for P4, being 79% higher in depressed zones than in elevated zones (Table 2 and Table 3).

**Figure 3:**
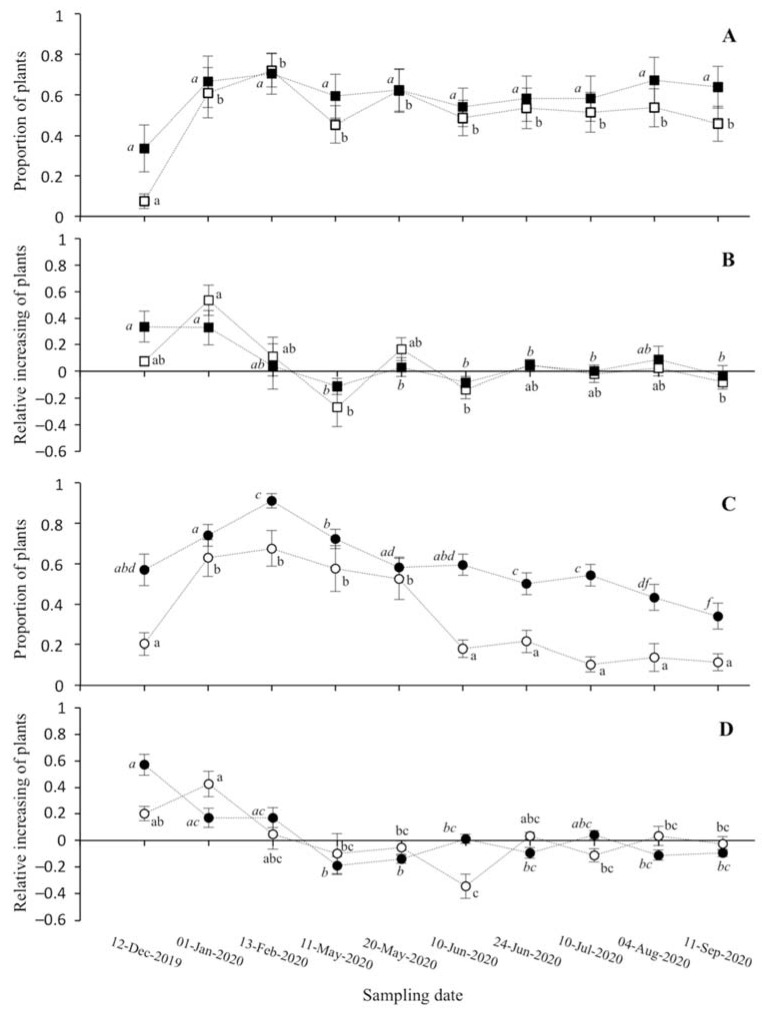
Density of plants (**A**,**C**) and increasing plant density (**B**,**D**) in relation to the maximum density reached for four populations of *Salicornia ramosissima* colonizing (**A**) elevated zones (Population 1, white squares; Population 2, black squares) and (**C**) depressed zones (Population 3, white circles; Population 4, black circles) from December 2019 to September 2020. Values are mean ± SE.

**Figure 4:**
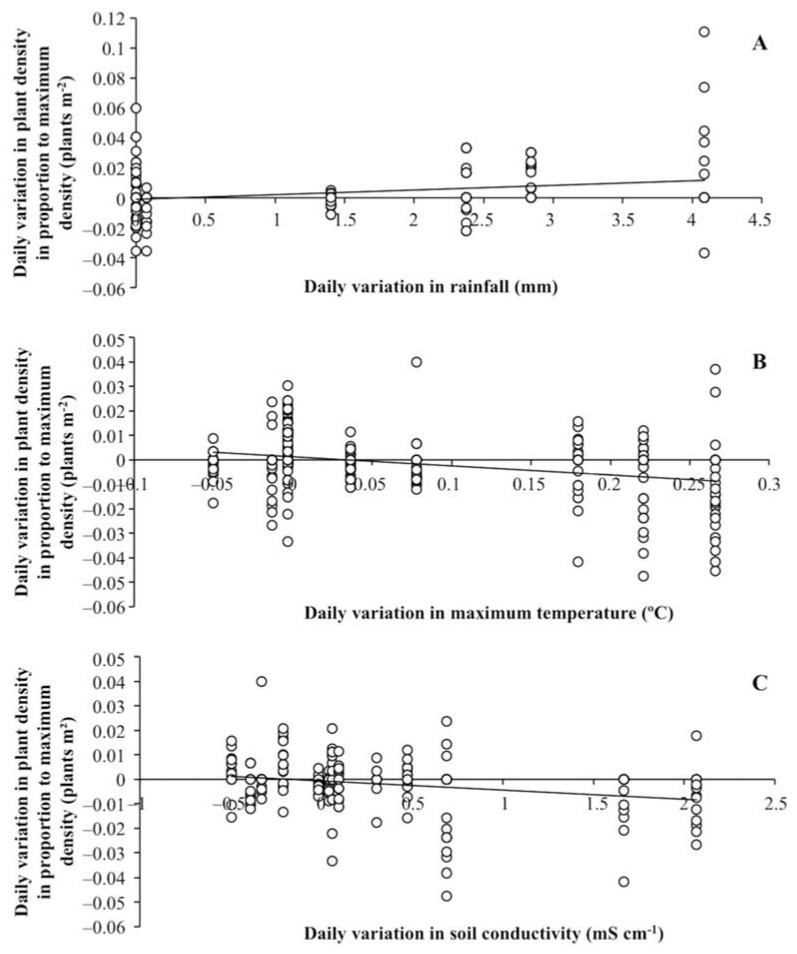
Relations between daily variation in plant density (plant m^−2^) in proportion to the maximum plant density and (**A**) daily variations in rainfall (mm) for elevated zones, (**B**) daily variations in maximum air temperature (°C) in depressed zones, and (**C**) daily variation in soil conductivity (mS cm^−1^) in depressed zones. Regression equations: (**A**) y = 0.0031x − 0.0010 (R^2^ = 0.084, *p* = 0.0058, *n* = 207); (**B**) y = −0.0372x + 0.0013 (R^2^ = 0.104, *p* = 0.0005, *n* = 270); (**C**) y = −0.0037x − 0.0006 (R^2^ = 0.066, *p* = 0.0218, *n* = 270).

The plant density increased from December to February for every *S. ramosissima* population, then the populations in elevated zones maintained constant values until flowering (Figure 3A), whereas plant density abruptly decreased (c. 60%) to the end of the study for the populations in depressed zones (Figure 3B). The daily variation in plant density in each sampling ring in proportion to the maximum plant density increased together with daily rainfall only in the elevated zones (Figure 4A, Table 4). In contrast, the daily variation in plant density decreased when the maximum air temperature and sediment EC increased in the depressed zones (Figure 4B,C, Table 4).

The maximum density of *S. ramosissima* plants varied between 1715 ± 256 plants m^−2^ for P2 and 18,947 ± 3683 plants m^−2^ for P4, being five-times greater in depressed zones than in elevated zones (Table 2 and Table 3). Thus, the maximum plant density increased together with soil-seed-bank density (ρ = 0.760, *p* = 0.0041, *n* = 12), representing between 14 ± 3% for P1 and 31 ± 17% for P2 of the soil-seed-bank density, with no significant differences among populations (Kruskal–Wallis test: H_(3,12)_ = 2.08, *p* = 0.5566).

**Figure 5:**
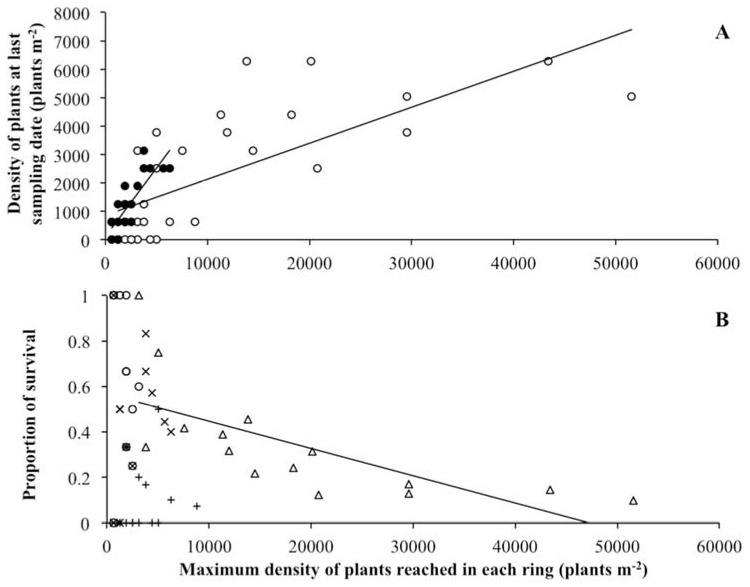
Relations between the maximum plant density reached in each sample ring and (**A**) plant density on the last sampling date in elevated zones (black circles) and depressed zones (white circles), and (**B**) the proportion of surviving plants in each sample ring in four *Salicornia ramosissima* populations (P1, cross; P2, circle; P3, plus; P4, triangle). Regression equations: (**A**) elevated zones, y = 0.482x + 108.790 (R^2^ = 0.688, *p* < 0.0001, *n* = 23); depressed zones y = 0.127x + 861.010 (R^2^ = 0.537, *p* < 0.0001, *n* = 30); (**B**) P4, y = −0.00001x + 0.567 (R^2^ = 0.473, *p* < 0.0001, *n* = 15).

The density of the surviving plants at the end of the study increased together with maximum plant density in elevated and depressed zones (Figure 5A, Table 5), showing similar values in both (Table 2 and Table 3). In relation to the soil-seed-bank density, the plant density at the end of the study represented 15 ± 12% for P1, 14 ± 4% for P2, 2 ± 1% for P3 and 4 ± 1% for P4, with no significant differences among populations (Kruskal–Wallis test: H_(3,12)_ = 5.36, *p* = 0.1473). The proportion of surviving plants in relation to the maximum plant density varied between 11 ± 4% for P3 and 64 ± 10% for P2, being 58% greater in elevated zones than in depressed zones (Table 2 and Table 3).

At the end of the study, the density of blooming and fruiting plants was similar in elevated and depressed zones. The proportion of blooming and fruiting plants in relation to the plant density at the end of the study was also similar between physiographic zones. In contrast, the proportion of blooming and fruiting plants in relation to the maximum plant density was 55% higher in elevated zones than in depressed zones (Table 2). Thus, the density of blooming and fruiting plants increased together with the maximum plant density in elevated zones and depressed zones (Table 5). Furthermore, the final proportions of surviving and blooming and fruiting plants decreased when the maximum plant density increased only for P4 (Figure 5B, Table 5). The plant height at the end of the study did not change between elevated and depressed zones (Table 2 and Table 3).

## 3. Discussion

Our results show that the annual halophyte *S. ramosissima* presents contrasted population dynamics depending on the physiographic position in salt pans under a Mediterranean climate, in relation to differences in plant density, partially in agreement with our first hypothesis, and to the environmental matrix, refuting our second hypothesis.

In elevated areas with good drainage, *S. ramosissima* populations presented maximum plant densities of between 628 and 6288 plants m^−2^ that remained almost constant until fruiting. These maximum densities maintained a dynamic equilibrium due to new seedling establishments and plant survival rates of between 46–64%. This survivorship curve pattern, which could be assimilated to Type I of [37], reveals that the mortality of individual plants was concentrated at the end of their lifespan. In contrast, *S. ramosissima* populations colonizing frequently waterlogged depressed zones presented five-times greater soil-seed-bank densities and maximum plant densities (between 1257 and 51,558 plants m^−2^) than populations in elevated zones. These high seed-bank densities may be the result of higher in situ seed production [8] and the transport of seeds from elevated to depressed zones. Previous studies have reported a positive correlation between soil-seed-bank density and plant density for *S. ramosissima* [8] and *S. europaea* [19]. In our context, populations in depressed zones lost c. 60% of their maximum plant densities from the end of spring and throughout summer. This is a hot, dry period in the Mediterranean climate, when increasing air temperatures are related to high evapotranspiration rates that result in high sediment salinities [7,38] and high plant mortality in salt marshes [39]. Thus, we began to record plant mortality from when the maximum air temperature increased by 0.05 °C or more daily, and from when the sediment EC started to increase. Rainfall and sediment moisture, and salinity, have been reported as the major environmental factors controlling establishment and death, respectively, in different *Salicornia* species [20,21,29,32,40]. The lowest EC recorded in our study was c. 10–20 mS cm^−1^ in winter and early spring, which correspond to salinities c. 200 mM NaCl that are close to the optimum growth range recorded for *S.* europaea (between 200–400 mM NaCl) [41]. The highest EC was c. 60–80 mS cm^−1^ in late spring and summer, corresponding to c. 600–800 mM NaCl, which are values that have been reported as growth-limiting for *S.* europaea (>400 mM NaCl) [41]. The survivorship curve pattern for *S. ramosissima* in the depressed zones could be assimilated to Types II or III of [34] and describes a situation in which individuals are affected by high mortality rates from the beginning of their adult stage. Similar curves were observed by [25] for populations of *S. ramosissima* with densities of 3000–9000 plants m^−2^ in salt pans in the Odiel Marshes. Moreover, [19] observed Type II surviving curves for *S. europaea* on the coast of Norfolk (England), stating that the proportion of plants that died before flowering depended on environmental conditions rather than on seedling density per se. Similarly, [33] indicated that abiotic stress was the primary cause of mortality in *S. europaea*, since its survival was not related to peaks in plant density as high as 65,000 plants m^−2^. In whatever way the environmental conditions seemed to control the population dynamic of *S. ramosissima* in depressed zones, the effects of a stressful environment would interact with plant densities since sediment EC and air temperatures were similar between physiographic locations, but plant densities were higher in depressed zones than in elevated zones. Along these lines, [42] registered a Type I survivorship curve for a density of 261 plants m^−2^ and a Type II survivorship curve for denser populations of *S. ramosissima* in the Aveiro Lagoon (Northwest Iberian Peninsula), proposing that the combined effect of sediment salinity and competition could cause the abrupt decrease in plant density observed in dense populations of *S. ramosissima* (c. 1700 plants m^−2^). Besides the contrasted population dynamics recorded in our study and that previous works have reported local adaptation to salinity for different *Salicornia* populations [43], in our case, it did not seem that there was local adaptation since the populations of elevated and depressed areas were very close to each other, so there would be genetic exchange between them, both through pollen and seeds.

Contrary to our first hypothesis, we found density-dependent mortality for the densest population (P4; max. 51,558 plants m^−2^). This result contradicts the density-independent seedling mortality recorded for *S. europaea* in Northern European marshes where the environmental matrix is more benign [19,22,23,24]. In this regard, [44] designed a field study on the North Atlantic Coast of North America comparing *S. europaea* responses at different individual densities, from 100 to more than 10,000 plants m^−2^, and concluded that plant density affected plant biomass and morphology, but not mortality. Thus, the general principle for halophyte survival states that mortality is mostly influenced by abiotic stresses rather than by plant densities [45]. However, some exceptions have already been established, such as *Spergularia marina* (L.) Griseb. [46].

Besides these contrasted responses in population dynamics that depend on physiographic position in the salt pans, all *S. ramosissima* populations concentrated the establishment of new seedlings during winter under a Mediterranean climate, when sediment salinity reached the lowest values. In this sense, *S. ramosissima* presents its maximum germination rates in fresh water after exposure to high salinities (0.6–0.9 M) [18]. Taking advantage of germination windows is especially important for halophytes colonizing highly stressful habitats such as salt pans [47,48,49]. In addition, air temperature plays an important role in the germination of *S. ramosissima* given that its seedling establishment occurred mostly from March to May in the Northwest Iberian Peninsula [42], where air temperatures are lower than in the Odiel Marshes. Low temperatures inhibit the germination of different *Salicornia* species [29,50]. On the other hand, maximum plant densities represented between 14–31% of the seed-bank density at both physiographic positions. [51] reported maximum plant densities representing between 10–59% of the seed-bank density for *S. procumbens* and c. 41% for *S. europaea* in The Netherlands. Our results show that *S. ramosissima* retained viable non-germinated seeds after the maximum peak of seedling establishment. In fact, we recorded increases in plant density during spring and summer after maximum plant densities were reached. This temporal germination strategy at the population level was probably based on a staggered breaking of the physiological dormancy induced by high salinities [17,18]. Therefore, this temporal pattern of germination may allow some *S. ramosissima* plants to avoid catastrophic events, such as herbivory or violent river floods, that could impact its populations throughout the growing season. Even so, the *S. ramosissima* seed bank was drastically reduced during the first year after seed dispersal [8], as occurred in *S. europaea* [19]. Additionally, all study populations presented similar plant height that was unrelated to plant density, so it seems to be linked to particular habitat characteristics or/and genetic differentiation, as demonstrated in *S. europaea* using transplant experiments [23,27,32].

In view of our results, climate change, which causes sea level rise [52] and reduces rainfall in the Mediterranean Basin [53], would increase sediment salinity in the salt pans thereby reducing the germination and establishment of *S. ramosissima*. This reduction in seedling establishment may reduce the final densities of fruiting plants in elevated zones and, at the same time, may lessen the density-dependent dynamic in depressed zones. In this scenario of climate change, halophytes offer an exceptional opportunity for saline agriculture [5]. Thus, our results are useful for planning the sustainable harvesting of natural populations of *S. ramosissima*. For example, plants could be harvested at the end of winter or the beginning of spring in depressed areas where plant densities would be greater than c. 15,000 plants m^−2^, which would help to avoid density-dependent mortality. Concrete harvest strategies should be designed for each population of *Salicornia* since they inhabit different environmental conditions and show high genetic and morphological variability [54]. In this context, our results are useful for the protection, restoration and sustainable exploitation not only of coastal salt marshes, but also of inland salt marsh habitats colonized by *Salicornia* species [55].

## 4. Materials and Methods

### 4.1. Study Area

The present study was carried out in tidal salt marshes of the Odiel Natural Park (37°12′32.3″ N, 6°58′01.5″ W) located in the Gulf of Cádiz (Southwest Iberian Peninsula). The Odiel Marshes are one of the largest areas of salt marshes in the Iberian Peninsula and are protected as a Natural Reserve of the Biosphere by UNESCO. The study area has a semi-diurnal mesotidal regime, with a tidal range (equinoctial mean) of 2.97 m [56]. The Odiel Marshes are subjected to a Mediterranean climate with Atlantic influence [57]. Annual mean air temperature is 18 °C and mean monthly temperatures range between +11 °C in January and + 25 °C in August. Annual average precipitation is 523 mm, with a 4–5 month dry period from approximately June to September (data series 1984–2010 from the meteorological station at Francisco Montenegro Avenue, in the city of Huelva, located close to the marshes under study (37°14′29″ N, 06°56′55″ W). We sampled four populations of *S. ramosissima* in two elevations in two different salt pans: two populations (P1 and P2) located in elevated areas that were rarely inundated, and another two populations (P3 and P4) colonizing depressed areas that were usually waterlogged. The elevation difference between these two physiographic positions was c. 20 cm. Previously, we characterized these two physiographic positions in another study on the *S. ramosissima* seed bank [8]. P1 and P3 were located in an abandoned saltwork evaporation pond (37°15′41.6″ N, 6°58′35.54″ W). P2 and P4 colonized salt pans (P2: 37°13′39.63″ N, 6°57′46.52″ W; P4: 37°13′34.74″ N, 6°57′50.54″ W).

### 4.2. Meteorological Data

Daily mean, maximum and minimum air temperatures and precipitation during the study period were obtained from the meteorological station at Francisco Montenegro Avenue. We calculated daily variations in air temperature for a certain sampling period as the temperature difference between two consecutive sampling dates divided by the number of days of that period. Daily rainfall for a sampling period was calculated by dividing the total rainfall accumulated since the previous sampling date by the number of days of the period.

### 4.3. Sedimentary Environment

We randomly choose three zones in each study population of *S. ramosissima*. In each zone, we took three sediment samples on 12 December 2019, 1 January, 13 February, 11 May, 10 June, 24 June, 10 July, 4 August and 11 September 2020, resulting in a total of 324 samples. Sediment samples were randomly collected using stainless-steel cores of 50 mm diameter and 50 mm height. Samples were placed in hermetically sealed polyethylene bags and stored at −20 °C until laboratory analysis. Sediment electrical conductivity (EC) and pH were measured in the unfiltered supernatant of a homogenized mix of 5 mL of wet sediments and the same volume of distilled water (1:1, *v*:*v*) using a conductivity meter, Crison Instruments 5064 (Hach Lange Spain, S.L.U., Barcelona, Spain), and a Crison pH meter 25 (Hach Lange Spain, S.L.U., Barcelona, Spain) [58]. We calculated daily variations in EC and pH for a sampling date by subtracting the value obtained in the previous sampling from the value obtained on the current date, divided by the number of days between these sampling dates. Field sediment salinity was calculated from electrical conductivity following this equation: Salinity (ppm Na Cl) = EC (μS cm^−1^) × 0.46 [59].

### 4.4. Soil Seed Bank of Salicornia ramosissima

The soil seed banks were studied at the beginning of the study, on 12 December 2019. We took 15 soil samples per population, 5 at each of three randomly selected zones, using stainless-steel cores of 50 mm diameter and 50 mm height. Samples were placed in hermetically sealed polyethylene bags and transported to the laboratory for analysis. Sediment samples were sieved through a 0.4 mm light sieve to eliminate most of the clay matrix, and the material that remained in the sieve was examined under a magnifying glass to look for seeds [60]. We calculated the density of seeds (seeds m^−2^) for each population.

### 4.5. Population Dynamic of Salicornia ramosissima

On 12 December 2019, we placed 15 plastic rings of 4.5 cm diameter around each *S. ramosissima* population, 5 at each of three randomly selected zones. These rings were labeled and anchored to the ground using stakes. Each *S. ramosissima* plant growing within each ring was counted on 12 December 2019, 1 January, 13 February, 11 May, 20 May, 10 June, 24 June, 10 July, 4 August and 11 September 2020. On 11 September 2020, we counted those plants that were blooming or presented ripening fruits and recorded the height of every *S. ramosissima* plant inside all the sampling rings, measuring from the sediment surface to the highest plant tip. Seven sampling rings in which no plant was observed during the study were discarded from analyses. Signs of predation on some parts of flowering or fruiting plants of *S. ramosissima* were only sporadically observed after the last sampling date.

We calculated the plant density per surface unit for each sampling ring at each sampling date. Then, we established the sampling date in which the highest density value was reached and the value of this maximum plant density. With these data for each sampling ring, we calculated the final proportion of surviving plants as plant density on the last sampling date (11 September 2020) in relation to the maximum plant density reached for each ring. We also calculated the proportion of blooming plants in relation to the total plant density on the last sampling date, and to the maximum plant density reached. In addition, we calculated the daily variation in plant density relative to the maximum density for each sampling ring on each sampling date, as the difference between the numbers of plants on two consecutive sampling dates divided by the maximum density reached and by the number of days between those sampling dates.

### 4.6. Data Analyses

Analyses were carried out using Statistica 8.0 (StatSoft INC., USA). Deviations from the mean were calculated as standard error (SE). Significant differences were considered when *p* < 0.05. Data or their transformations (log (x + 1), 1/(x + 1) and √x) were tested for homogeneity of variance and normality with the Levene test and the Kolmogorov–Smirnov test, respectively. None of the tested data series followed normal distribution; therefore, the Kruskal–Wallis test was used to compare significant differences between the means of the four study populations; the Mann–Whitney U test was applied to detect significant differences between the means in elevated and depressed areas. The non-parametric Spearman’s correlation coefficient (ρ) was used to analyze the relationships between sedimentary variables and the recorded variables for *S. ramosissima* plants.

## Figures and Tables

**Table 1:** Sediment pH and electrical conductivity (EC) (mS cm^−1^) and seed-bank density (seed m^−2^) for four populations of *Salicornia ramosissima*, and in elevated and depressed zones. Different letters indicate significant differences among populations (Kruskal–Wallis test, *p* < 0.05) or between elevated and depressed zones (Mann–Whitney test, *p* < 0.05). Data are mean ± SE.

	1	2	3	4	Elevated Zones	Depressed Zones
Soil seed bank (seed m^−2^)	23,474 ^a^ ± 9595 (*n* = 15)	7671 ^a^ ± 1767 (*n* = 15)	27,162 ^ab^ ± 6356 (*n* = 15)	124,620 ^b^ ± 30,825 (*n* = 15)	15,572 ^A^ ± 5013 (*n* = 30)	75,891 ^B^ ± 17,916 (*n* = 30)
Soil pH	6.8 ^a^ ± 0.1 (*n* = 81)	6.5 ^c^ ± 0.1 (*n* = 81)	7.0 ^b^ ± 0.1 (*n* = 81)	6.7 ^a^ ± 0.0 (*n* = 81)	6.6 ^A^ ± 0.0 (*n* = 162)	6.8 ^B^ ± 0.0 (*n* = 162)
Soil conductivity (mS cm^−1^)	54.7 ^a^ ± 5.0 (*n* = 81)	35.9 ^b^ ± 3.3 (*n* = 81)	45.5 ^ab^ ± 3.9 (*n* = 81)	32.7 ^b^ ± 2.0 (*n* = 81)	45.3 ^A^ ± 3.1 (*n* = 162)	39.1 ^B^ ± 2.2 (*n* = 162)

**Table 2:** Kruskal–Wallis test and Mann–Whitney U-test comparing between four populations of *Salicornia ramosissima*, two salt-pan elevations and nine sampling points for sedimentary and plant variables.

	Between Populations	Between Elevations	Between Sampling Dates
Sediment pH	H_(3,324)_ = 49.75, *p* < 0.0001	U = 2886.0, *p* < 0.001	P1: H_(8,81)_ = 54.92, *p* < 0.0001; P2: H_(8,81)_ = 56.39, *p* < 0.0001; P3: H_(8,81)_ = 64.90, *p* < 0.0001; P4: H_(8,81)_ = 61.35, *p* < 0.0001
Sediment electrical conductivity (mS cm^−1^)	H_(3,324)_ = 10.91, *p* = 0.012	U = 3847.5, *p* = 0.562	P1: H_(8,81)_ = 71.09, *p* < 0.0001; P2: H_(8,81)_ = 65.24, *p* < 0.0001; P3: H_(8,81)_ = 65.07, *p* < 0.0001; P4: H_(8,81)_ = 63.63, *p* < 0.0001
Seed-bank density (seeds m^−2^)	H_(3,60)_ = 24.22, *p* <= 0.0001	U = 160.0, *p* < 0.0001	-
Maximum plant density (plants m^−2^)	H_(3,53)_ = 29.53, *p* < 0.0001	U = 115.0, *p* < 0.0001	-
Density of surviving plants at the end of the study (plants m^−2^)	H_(3,53)_ = 34.22, *p* = 0.0000	U = 281.5, *p* = 0.246	-
Density of fruiting plants (plants m^−2^)	H_(3,53)_ = 34.22, *p* < 0.0001	U = 273.5, *p* = 0.190	-
Proportion of fruiting plants in relation to plant density at the end of the study	H_(3,53)_ = 1.51, *p* = 0.6802	U = 186.0, *p* = 0.524	-
Proportion of fruiting plants in relation to maximum plant density	H_(3,53)_ = 18.20, *p* = 0.0004	U = 174.0, *p* = 0.002	-
Proportion of surviving plants in relation to maximum plant density	H_(3,53)_ = 19.28, *p* = 0.0002	U = 145.0, *p* = 0.0002	-
Plant height (cm)	H_(3,53)_ = 15.83, *p* = 0.0012	U = 200.5, *p* = 0.622	-

**Table 3:** Population parameters for four populations of *Salicornia ramosissima*, and for populations in elevated and depressed zones. Different letters indicate significant differences among populations (Kruskall–Wallis test, *p* < 0.05) or between physiographic zones (Mann–Whitney test, *p* < 0.05). Values are mean ± SE.

	1	2	3	4	Elevated Zones	Depressed Zones
Mean sampling date (in ordinal number) when maximum plant density was reached	3.75 ^a^ ± 0.60 (*n* = 12)	3.55 ^a^ ± 0.88 (*n* = 11)	3.13 ^a^ ± 0.26 (*n* = 15)	2.73 ^a^ ± 0.23 (*n* = 15)	3.65 ^A^ ± 0.51 (*n* = 23)	2.93 ^A^ ± 0.17 (*n* = 30)
Mean maximum plant density reached (plant m^−2^)	2777.0 ^ab^ ± 565.9 (*n* = 12)	1714.8 ^b^ ± 255.6 (*n* = 11)	3521.1 ^a^ ± 541.23 (*n* = 15)	18,946.6 ^c^ ± 3682.6 (*n* = 15)	2269.00 ^a^ ± 332.47 (*n* = 23)	11,233.82 ^b^ ± 2322.81 (*n* = 30)
Mean plant density at last sampling date (plant m^−2^)	1362.3 ^a^ ± 335.9 (*n* = 12)	1028.9 ^a^ ± 175.25 (*n* = 11)	419.2 ^b^ ± 169.9 (*n* = 15)	4149.8 ^c^ ± 377.3 (*n* = 15)	1202.84 ^A^ ± 193.30 (*n* = 23)	2284.49 ^A^ ± 401.62 (*n* = 30)
Mean proportion of survival plants (plant density at last sampling date respect to maximum plant density reached)	0.46 ^ac^ ± 0.09 (*n* = 12)	0.64 ^a^ ± 0.10 (*n* = 11)	0.11 ^b^ ± 0.04 (*n* = 15)	0.34 ^c^ ± 0.06 (*n* = 15)	0.54 ^A^ ± 0.07 (*n* = 23)	0.23 ^B^ ± 0.04 (*n* = 30)
Mean density of blooming plants at last sampling date (plant m^−2^)	1152.7 ^a^ ± 335.9 (*n* = 12)	857.4 ^a^ ± 153.4 (*n* = 11)	335.3 ^b^ ± 135.4 (*n* = 15)	4066.0 ^c^ ± 372.6 (*n* = 15)	1011.48 ^A^ ± 188.50 (*n* = 23)	2200.66 ^A^ ± 397.38 (*n* = 30)
Mean proportion of blooming plants at last sampling date respect to plant density at last sampling date	0.83 ^a^ ± 0.12 (*n* = 10)	0.87 ^a^ ± 0.07 (*n* = 10)	0.96 ^a^ ± 0.04 (*n* = 6)	0.98 ^a^ ± 0.01 (*n* = 15)	0.85 ^A^ ± 0.07 (*n* = 20)	0.97 ^A^ ± 0.02 (*n* = 21)
Mean proportion of booming plants at last sampling date respect to maximum plant density reached	0.39 ^a^ ± 0.10 (*n* = 12)	0.54 ^a^ ± 0.10 (*n* = 11)	0.08 ^b^ ± 0.03 (*n* = 15)	0.33 ^a^ ± 0.06 (*n* = 15)	0.47 ^A^ ± 0.07 (*n* = 23)	0.21 ^B^ ± 0.04 (*n* = 30)
Mean height of plants at last sampling date	6.40 ^a^ ± 0.94 (*n* = 10)	12.92 ^b^ ± 0.96 (*n* = 10)	11.79 ^abc^ ± 3.17 (*n* = 7)	8.17 ^c^ ± 0.52 (*n* = 15)	9.66 ^A^ ± 0.99 (*n* = 20)	9.32 ^A^ ± 1.08 (*n* = 22)

**Table 4:** Spearman correlation coefficient (ρ) and probability value (*p*) for correlations between daily variation in plant density in proportion to maximum density (plants m^−2^), and daily variation in different environmental variables for four populations of *Salicornia ramosissima* and for populations in elevated and depressed zones. Significant values are marked in bold (*p* < 0.05).

Maximum Plant Density vs.		1	2	3	4	Elevated Zones	Depressed Zones
Plant density at last sampling date	r	**0.823**	0.579	0.404	**0.542**	**0.801**	**0.811**
	*p*	**0.0010**	0.0618	0.1349	**0.0368**	**0.0000**	**0.0000**
Proportion of survival plants	r	0.133	−0.389	0.221	**−0.897**	−0.092	0.223
	*p*	0.6803	0.2372	0.4293	**0.0000**	0.6748	0.2371
Density of blooming plants at last sampling date	r	**0.685**	0.600	**0.521**	**0.543**	**0.693**	**0.829**
	*p*	**0.0140**	0.0512	**0.0463**	**0.0364**	**0.0002**	**0.0000**
Proportion of blooming plants respect to maximum plant density reached	r	0.032	−0.350	0.397	**−0.908**	−0.126	0.335
	*p*	0.9211	0.2913	0.1421	**0.0000**	0.5664	0.0703
Mean height of plants at last sampling date	r	−0.518	0.222	0.019	0.098	−0.348	0.022
	*p*	0.1255	0.5370	0.9683	0.7274	0.1332	0.9242

**Table 5:** Spearman correlation coefficient (ρ) and probability value (*p*) for correlations between the maximum plant density reached in each sampling ring and different population and plant characteristics for four populations of *Salicornia ramosissima*, and for populations in elevated and depressed zones. Significant values are marked in bold (*p* < 0.05).

Daily Variation in Plant Density in Proportion to Maximum Density vs.		1	2	3	4	Elevated Zones	Depressed Zones
Daily rainfall	r	**0.283**	0.083	**0.198**	0.020	**0.191**	0.098
	*p*	**0.0030**	0.4172	**0.0214**	0.8177	**0.0058**	0.109
Daily variation in maximum temperature	r	−0.020	−0.109	**−0.344**	−0.102	−0.061	**−0.210**
	*p*	0.8376	0.2851	**0.0000**	0.2399	0.3825	**0.0005**
Daily variation in minimum temperature	r	0.084	−0.012	**−0.259**	0.013	0.039	−0.118
	*p*	0.3876	0.9058	**0.0025**	0.8811	0.5761	0.0531
Daily variation in soil pH	r	−0.087	0.034	−0.037	**−0.223**	−0.017	−0.128
	*p*	0.4317	0.7692	0.7058	**0.0222**	0.8314	0.0636
Daily variation in soil conductivity	r	−0.070	0.060	**−0.230**	**−0.194**	−0.016	**−0.158**
	*p*	0.5274	0.6021	**0.0181**	**0.0471**	0.8411	**0.0218**

## Data Availability

The data presented in this study are openly available in FigShare. Available online: https://doi.org/10.6084/m9.figshare.19960142 (accessed on 2 June 2022).
